# Ras Pathways on Prox1 and Lymphangiogenesis: Insights for Therapeutics

**DOI:** 10.3389/fcvm.2020.597374

**Published:** 2020-11-12

**Authors:** Khoa Bui, Young-Kwon Hong

**Affiliations:** Department of Surgery, Department of Biochemistry and Molecular Medicine, Norris Comprehensive Cancer Center, Keck School of Medicine, University of Southern California, Los Angeles, CA, United States

**Keywords:** lymphangiogenesis, Prox1, Ras, PI3K-AKT pathway, ERK pathway, lymphatics

## Abstract

Over the past couple of decades, lymphatics research has accelerated and gained a much-needed recognition in pathophysiology. As the lymphatic system plays heavy roles in interstitial fluid drainage, immune surveillance and lipid absorption, the ablation or excessive growth of this vasculature could be associated with many complications, from lymphedema to metastasis. Despite their growing importance in cancer, few anti-lymphangiogenic therapies exist today, as they have yet to pass phase 3 clinical trials and acquire FDA approval. As such, many studies are being done to better define the signaling pathways that govern lymphangiogenesis, in hopes of developing new therapeutic approaches to inhibit or stimulate this process. This review will cover our current understanding of the Ras signaling pathways and their interactions with Prox1, the master transcriptional switch involved in specifying lymphatic endothelial cell fate and lymphangiogenesis, in hopes of providing insights to lymphangiogenesis-based therapies.

## Introduction

The lymphatic system is a vascular system that shadows the well-known cardiovascular or circulatory system. The circulatory system plays roles in delivering essential nutrients, hormones, and oxygen across the body, where fluid extravasates from the arterial ends of capillary beds to transport these components to the surrounding tissue, and then gets reabsorbed into the venous ends of the capillary beds to return to the venous circulation. However, ~10% of this fluid is unable to be reabsorbed, due to the circulatory system's overall higher capillary hydrostatic pressure and lower blood colloidal osmotic pressure (1, 2). To compensate for this loss, the lymphatic system facilitates the return of remaining interstitial fluid by draining it through lymphatic vessels and lymph nodes as lymph, which eventually returns to the circulatory system via the subclavian veins. In addition to this drainage role, the lymphatic system is involved in immunosurveillance, serving as a centralized hub for activating naïve B and T lymphocytes via antigen-presenting cells that drain through the lymph (3). Furthermore, the lymphatic system facilitates fatty acid absorption from the digestive system, where lymph vessels that line the intestines, lacteals, take up chylomicrons and take them to the blood circulation for downstream processing (4).

Lymphangiogenesis is the generation and sprouting of lymphatic endothelial cells (LECs) from preexisting ones, mirroring that of angiogenesis (5, 6). This process is the major mode of lymphatic growth and is essential to the development of the lymphatic system during embryogenesis. Lymphangiogenesis revolves around the transcription factor Prospero Homeobox 1, Prox1 (7). The formation of the lymphatic vasculature begins with Prox1 expression in a subset of blood endothelial cells (BECs) in the cardinal vein, where these Prox1-expressing cells ultimately bud off and migrate toward vascular endothelial growth factor C (VEGF-C) to create a lymph sac, which forms into primary lymphatic plexus and matures as the lymphatic network (8). Lymphangiogenesis gives rise to the complete lymphatic system and is also involved in disease response, though there are studies that suggest that other cells could transdifferentiate into LECs (9, 10). Outside of embryonic development, lymphangiogenesis may be induced following injury to assist in wound healing. For example, skin wound healing studies have shown increases in lymphatic vessel density and quicker recovery times as opposed to those with impaired lymphangiogenesis (11). The past several years has also presented new findings on the ability of cardiac lymphangiogenesis to reduce myocardial edema and fibrosis following cardiac injury (12).

Given the lymphatic system's roles in fluid homeostasis and immunity, lymphangiogenesis is governed by multiple signaling pathways in both development and pathophysiological responses through different manners, given the contrasting microenvironment of these two models. As such, levels of growth factors and inflammatory cytokines play significant and unique roles in controlling vascular growth. A moderate balance of lymphatic vasculature must be maintained; the lack of mature as well as the excess of immature lymph vessels can impair lymphatic function, where vessels become “leaky” and are unable to properly transport lymph. This has been highlighted through numerous knockout studies, revealing key pathways involved in this process (Table 1). The dysregulation of lymphangiogenesis through the inhibition or over-stimulation of signaling pathways often leads to lymphatic vessel malfunction.

**Table 1:** Knockout mouse models and associated lymphatic phenotypes.

**Gene of Interest**	**Mutant Mouse Models and Observations**	**Proposed function**
**Lymphatic Phenotypes Reported in Knockout Mouse Models**		
*Prox1*	A) Global *Prox1^+/−^*: Reduction in LEC number followed with the loss of lymphovenous valves (13). Severe edema is detected at mid-gestation stage at E14.5. Most die within 2–3 days after birth, presenting chylous ascites (14). Surviving mice maintain impaired lymphatic vasculature and encounter adult-onset obesity (15) B) *Tie2-Cre; Prox1 ^*fl*/+^*: Same as A C) Global *Prox1^−/−^*: LEC specification lost and budding/sprouting arrested around E11.5, shortly after initiation; no lymphatic vasculature (7). Mice present severe edema and die around mid-gestation stage at E14.5–15 (7, 14)	Prox1 is a master switch for LEC specification, maintenance as well as sprouting
*Vegfc*	A) Global *Vegfc^+/−^*: Embryo lymph sac formation was normal or slightly reduced. Severe lymphatic vessel hypoplasia accompanied by chylous ascites in pups, with some recovery in first postnatal weeks. Cutaneous lymphatic vessel hypoplasia remained in skin of adults (16) B) Global *Vegfc^−/−^*: LECs unable to sprout and form lymphatic vessels. Severe edema from E12.5 onward. Embryonic death with half of embryos dying E15.5–E17.5. No live-born pups (16)	Key growth factor for Vegfr3 and thereby AKT and ERK signaling
*Vegfr3*	A) Global *Vegfr3^+/−^*: Embryos appear phenotypically normal (17). Lymphedema due to hypoplastic cutaneous lymphatic vessels (18) B) Global *Vegfr3^−/−^*: Embryonic death beginning at E10.5, prior to lymphatic vessel emergence/sprouting. No live-born pups (17). Abnormal vasculature, enlarged vascular bed formation, severe anemia before embryonic death (19)	Receptor for growth factors to initiate AKT and ERK signaling almost exclusively in LECs, though present in other ECs during early development
*Foxc1/2*	A) Global *Foxc1^−/−^*: Increased LEC specification and proliferation. Enlarged lymph sacs at E12.5. Subcutaneous edema from E15.5 onward with lymphatic vessel hyperplasia (20) B) *Prox1CreER^*T*2^; Foxc1^*fl*/*fl*^:* Same as A C) *Prox1CreER^*T*2^; Foxc2^*fl*/*fl*^* Same as A D) *Prox1CreER^*T*2^;Foxc1^*fl*/*fl*^/Foxc2^*fl*/*fl*^* Similar to A, but with ~5% rate of severe edema at E15.5 (20)	Foxc1 promotes the expression of GTPase-activating proteins that are coded by *Rasal3* and *Rasa4* to regulate the ERK pathway (20)
*Ras*	A) Global *Ras* TKO: Decreased lymphatic specification. Embryonic lethality B) Global *Ras* DKO: Pups presented chylous ascites and lymphatic vessel hypoplasia (21)	Ras is a major effector protein involved in AKT and ERK signaling (21)
*Rasa1*	A) *Ub-CreER^*T*2^; Rasa1^*fl*/*fl*^*: Following tamoxifen injection at 2 months of age, mice presented chylous ascites, lymphatic vessel dilation and extensive lymphatic vessel hyperplasia. All mice died by 8 months after tamoxifen administration (22)	*Rasa1* codes a negative regulator of vascular growth (22). *Rasa1* may modulate AKT and ERK signaling by turning off Ras
*Akt1*	A) Global *Akt1^−/−^*: Enlargement of collecting lymphatic vessels, increased number of LECs, sparse coverage of smooth muscle cells. Loss of valves in small collecting lymphatics in bottom side of ear skin (23)	*Akt1* a protein kinase that acts as a major signal transducer involved in both blood and lymphatic vascular development (23)
*Spred-1/2*	A) Global *Spred-1^−/+^/Spred-2^−/+^*: Mice are healthy and fertile (24) B) Global *Spred-1^−/−^/Spred-2^−/−^*: Embryonic death between E12.5–15.5 with severe subcutaneous hemorrhage, edema, and dilated lymph vessels. Blood vessels appear almost normal when compared to controls (24) C) Global *Spred-1^−/−^/Spred-2^−/+^*:Similar bleeding as A. Mice rarely born and most died within a few months (24) D) Global *Spred-1^−/+^/Spred-2^−/−^*:Mice were healthy and fertile, but smaller than mice described in A (24)	Spred-1 and Spred-2 are negative regulators in VEGF-C/VEGFR-3 signaling, inhibiting ERK and AKT activity (24)
*Cdh5*	A) *Prox1CreER^*T*2^; Cdh5^*fl*/*fl*^*: Following tamoxifen injection at E10: Dilated lymphatic vessels in mesentery, valves still absent by E18.5. Edema observed at E14.5 and onward. Pups presented chylous ascites (25) B) Global *Cdh5^−/−^*: Embryonic death at E9.5 (26)	VE-Cadherin required for response to fluid shear stress and thereby Beta-Catenin and AKT signaling, promoting Prox1 and Foxc2 expression (25)

*These models tend to lead to loss of proper vascular development, typically manifested as edema in varying severity with the potential for embryonic death*.

Lymphatic vessel malfunction is associated with the pathogenesis of many diseases, including inflammatory disease (27, 28), lymphedema, or tumor-associated lymphangiogenesis (29). The loss of lymphatic function could lead to impaired fluid drainage and immunosurveillance capability during disease, exacerbating the pathological conditions that exist. Inhibiting fetal lymphangiogenesis through VEGF-C sequestration has been shown to lead to lymphedema (30). In contrast, increased lymphangiogenesis enables cancer metastasis similar to that of angiogenesis, where tumor cells are able to invade and travel through the vasculature (31). Interestingly, a major transcription factor of LECs, Prox1, was found to be overexpressed in multiple cancers, promoting not only lymphangiogenesis but also cancer cell migration capacity as well as invasiveness (32–34).

This review will look to cover our current understanding on both Prox1 and the major signaling pathways of lymphangiogenesis, so that we could better understand how they are tied in this process.

## The Lymphatic System's Role in Cancer

Like that of angiogenesis, lymphangiogenesis could open routes for cancer metastasis, where tumor cells may separate from a primary tumor and find their way to distant organs through these vascular systems. The significance of lymphatic vessels in metastasis was recognized through the usage of photodynamic therapy; the photodynamic ablation of these vessels and intralymphatic cancer cells prevented metastasis (35). This study highlighted that targeting the lymphatics is just as important as targeting the cardiovascular system to combat metastatic cancer. The lymphatic system was found to be directly involved in metastasis through the release of VEGF-C and other related growth factors by malignant tumors (36). Through the release of these growth factors, the lymphatic vasculature begins to sprout toward the tumor, which is then followed by the metastatic process. This metastatic process is comprised of multiple steps: (1) Invasion of primary tumor into surrounding tissue and basement membrane, (2) intravasation of tumor cells into surrounding vessels, (3) circulating tumor cells (CTCs) survival in the circulation, (4) CTCs arrest and extravasation at a distant organ site, and (5) metastatic formation/colonization of the site (37).

Besides metastasis, both vascular systems possess additional roles that support tumor growth. The cardiovascular system's roles in delivering oxygen and nutrients are vital to tumor growth, whereas the lymphatic system may help dampen anti-tumor immunity. Indeed, these lymphatic vessels do not just act as passive routes for metastasis, but are involved in immune modulation and cancer stem cell survival (36). This role may appear paradoxical, as the lymphatics are crucial for the initiation of tumor-specific T cell responses as seen in melanomas (38). However, there exists a negative feedback loop between LECs and cytotoxic T cells, where LECs activated by interferon gamma (IFN-γ) upregulate their expression of programmed death-ligand 1 (PD-L1), an immunosuppressive molecule that inhibits cytotoxic T cell accumulation in tumors (39). As such, the tumor microenvironment may abuse the expression of these inflammatory cytokines to promote their progression, generating an environment that may be intolerable to normal cells but not to the tumor.

In addition to impairing the adaptive immune response, it may be possible that increased lymphatic drainage would promote the clearance of waste products that are generated with the rapid growth and proliferation of cancer cells. Furthermore, it has been reported that lymph flow toward the sentinel nodes are increased, leading to mechanical stress-induced changes in stromal cells and thereby tumor microenvironment (40). This microenvironment would likely promote the growth of more vessels. However, there is a lot of controversy over the significance of intratumoral lymphatic vessels, as imaging studies have suggested that these vessels may be collapsed and non-functional as a result of high interstitial pressure within the tumor (41, 42). The disruption of this balance in interstitial fluid pressure is well-known to complicate drug delivery; high interstitial fluid pressure in most solid tumors impair the extravasation of therapeutic agents in the blood to the target site (43). These phenotypes are not just apparent in cancer; many diseases of excessive lymphatic remodeling have been met with lymphatic insufficiency, where the surrounding tissues of these leaky vessels are flooded.

## PROX1: A Powerful Regulator in Many Tissues

Prox1 is often referred to as the master switch for lymphatic endothelial cell (LEC) specification and sprouting, being a vital marker for LECs. However, Prox1 is not restricted to the lymphatic endothelium alone; it has a major role in pushing hepatoblasts toward the hepatocyte phenotype in liver, regulating neurogenesis, promoting the development of the heart, and so on (44–47). With these findings, Prox1 can be recognized as a cell fate switch in these tissues, playing a large role in cell differentiation. Even so, it is important to understand that the sets of genes that are induced or repressed by this transcription factor are cell type-specific; for example, Prox1 is found to promote the shift of colorectal cancer from benign to highly dysplastic, despite the lack of overlap between Prox1-induced genes in LECs vs. these colorectal cancer cells (48).

With advances in both lymphatic and cardiovascular research, it has been reported that there are unique vascular beds that possess a heterogenous expression of blood and lymphatic vessel markers, leading to the characterization of “hybrid vessels.” These specialized hybrid vessel beds include the Schlemm's canal of the eye, the placental spiral artery, and so on. Schlemm's canal, which is a vascular structure in the eye that drains aqueous humor from the intraocular chamber back into the circulatory system, acquires lymphatic characteristics through Prox1 upregulation during postnatal development (49). Along with this determination of endothelial identity, Prox1 levels linearly correlate with Schlemm's canal function, where reduced levels indicated poor functionality (49). Recent discoveries have identified Prox1's involvement in placental spiral artery remodeling; Prox1 begins to be expressed at E11.5 in the spiral artery endothelium of mice to promote lymphatic mimicry (50). Spiral arteries are used to supply maternal blood over to the fetal side of the placenta and thereby the fetal vasculature, with poor spiral artery remodeling being associated with pregnancy complications such as preeclampsia (51, 52). This dual expression of LEC and BEC markers can be seen across hybrid vessels, with Prox1 as a driver for these other LEC markers.

The aberrant expression of Prox1 has highlighted its role in endothelial cells (ECs). Its ectopic expression in blood endothelial cells (BECs) leads to an upregulation of lymphatic-specific genes, suggesting Prox1 is sufficient to program LECs (53). Ectopic Prox1 expression in human umbilical vein endothelial cells (HUVECs) and siRNA-mediated knockdown in LECs also revealed angiopoietin-2, forkhead box protein c2 (Foxc2), and homeobox D8 (HoxD8) as Prox1's targets for transcription (54). Prox1 knockouts in mice result in a loss of lymphatic markers such as lymphatic vessel endothelial hyaluronic acid receptor (LYVE-1), vascular endothelial growth factor receptor 3 (VEGFR-3) and the solute-carrier gene (SLC) superfamily, while gaining an expression of vascular markers such as laminin and CD34 (7). Indeed, Prox1 is able to induce LEC-specific gene transcription while suppressing BEC-specific genes (55). In addition to differential gene expression, knockout mouse models of transcription factor Prox1 presented complete or partial loss of lymphatic vasculature, resulting in death or a multitude of complications (14), including adult-onset obesity (15). Altogether, these findings suggest that Prox1 is not only necessary for LEC determination, but also lymphangiogenesis.

Prox1 can regulate the transcription of many genes through direct promoter binding. The Prox1 homeodomain consists of the characteristic helix-loop-helix-turn-helix fold structure that works together with the prospero domain to form a functional DNA-binding unit (56–58). Prox1 binds to the promoter of fibroblast growth factor receptor (FGFR-3) in LECs, inducing its transcription to support lymphatic vessel development (59). In contrast, Prox1 binds to matrix metallopeptidase 14 (MMP-14) promoter to repress its transcription (60). This finding suggests tumor suppressive roles for Prox1 with cancer invasion, though its role in cancer is context and tumor type-dependent; Prox1 been shown to play oncogenic roles in oral squamous cell carcinoma, for example (61, 62).

In addition to modulating transcription via direct DNA-binding, Prox1 may regulate gene expression through corepressor/coactivator activity. These interacting proteins range from a number of nuclear receptors to chromatin modifiers. The N-terminal end of Prox1 possesses a nuclear localization signal and two nuclear receptor boxes (61). In the liver, Prox1 plays a major role in energy homeostasis by limiting cellular respiration rate; Prox1 possesses LXXLL interaction motifs that allows for its interaction with liver receptor homolog 1 (LRH1) as a corepressor (63, 64) and can inhibit ERRα/PGC-1α complex activity (65). It also co-regulates hepatocyte nuclear factor 4 alpha (HNF4α) transcriptional activity of cholesterol catabolizing enzymes (66). Furthermore, Prox1 functions as a corepressor of the retinoic acid-related orphan receptors (RORs) by interacting with their activation function 2 (AF2) domain, though their interactions are independent of these LXXLL motifs (67). Retinoic acid signaling pathways are known for their anti-proliferative and pro-apoptotic effects, serving as potential chemotherapeutic approaches to cancer (68). Prox1 may be promoting proliferation in cancer through the inhibition of these retinoic acid signaling pathways.

Prox1 is also involved with epigenetic mechanisms that involve chromatin modification, such as histone methylation and acetylation. In lens development, Prox1 has been reported to interact with coactivator cAMP response element-binding protein (CREB)-binding protein (CBP) and/or p300 to upregulate crystallin gene expression via euchromatin formation (69). In contrast, histone deacetylase 3 (HDAC3)-Prox1 complexes were found to mediate a gene expression program important for lipid synthesis and lipolysis, where loss of either protein resulted in increases of liver triglyceride content (70). In the liver, Prox1 was also found to recruit lysine-specific demethylase 1 (LSD1) and HDAC2 to the cytochrome p450 family 7 subfamily A member 1 (CYP7A1) promoter, epigenetically silencing its transcription (71). In the context of colorectal cancer, Prox1 was found to interact with the nucleosome remodeling and deacetylase (NuRD) complex to suppress Notch signaling, thereby allowing these cancer cells to maintain their stem cell properties and growth advantage (72). This would further explain the differences in gene targets between LECs and colorectal cancer, as previously mentioned. Ultimately, these studies support the notion that Prox1 possesses roles dependent on tissue/organ function and disease context.

## VEGFR-3, A “Master Receptor” for lymphangiogenesis

Vascular endothelial growth-factor receptor (VEGFR) signaling regulates vascular function of both the cardiovascular and lymphatic system, where its three different types have varying roles across both systems. Whereas, VEGFR-2 is highly expressed in blood endothelial cells and is thereby crucial for angiogenesis, VEGFR-3 is required for the development of lymphatic vessels and is also important in early cardiovascular development, as VEGFR-3 is present in BECs at early embryogenesis (73–75). During later embryonic development, VEGFR-3 expression is largely restricted to LECs (14, 76). VEGFR-1 is primarily expressed during hematopoietic cell development and recruitment (77). While these three tyrosine receptor kinases have distinct functions in separate tissue compartments, they may all converge to promote pathological vessel formation in lymphatic diseases and tumor-associated lymphangiogenesis (78).

These tyrosine receptor kinases bind to the VEGF family of homodimeric glycoproteins, which consists of five members in mammals: VEGF-A, VEGF-B, VEGF-C, VEGF-D, and placental growth factor (PLGF). This group belongs to the cystine-knot superfamily of hormones and extracellular signaling molecules, which all possess eight conserved cysteine residues that form this knot. VEGFR-3 binds to VEGF-C (79) and VEGF-D, stimulating lymphangiogenesis (80). VEGF is produced by a number of cell types, including macrophages, keratinocytes, and tumor cells (81–83). Besides vascular development, VEGF plays roles in bone formation, hematopoiesis and wound healing (84–86). With regards to wound healing, ongoing developments on the cardiac lymphatics have highlighted their roles in the resolution of inflammation; VEGF-C treatment following myocardial ischemia and/or infarction allows for increased lymphatic drainage capacity of excess proteins (e.g., pro-inflammatory mediators), immune cells (e.g., macrophages) and fluid, which ultimately promotes cardiac function (87–89). Lymphatic vessels were found to have increased diameter with higher VEGF-C doses (12).

Where Prox1 is often referred to as a master switch in specifying LEC fate, VEGFR-3 could be viewed as a master receptor for LEC sprouting and migration. Knockout studies on VEGFR-3 and its ligands helped to define their distinct spatiotemporal roles in vessel formation, presenting a few differences. While heterozygous *Vegfr3* mice and embryos appeared phenotypically normal, complete knockouts of the *Vegfr3* gene results in pericardial effusion and cardiovascular failure by E9.5 (17). These findings explain the importance of VEGFR-3 during cardiovascular development, prior to their restriction to lymphatic vessels.

On the other hand, *Vegfc*^−/−^ mice were found to have endothelial cells commit to LEC fate as usual, but these cells were unable to sprout and form lymph vessels, resulting in prenatal death due to fluid accumulation (16). Half of these mutant embryos were found to die between E15.5 and E17.5 (16), which contrasts with the time of death of *Prox1* null mice, which primarily takes place at E14.5 (14). These deaths are likely attributed to a combination of phenotypic alterations, though the lymphatic phenotype likely plays a significant role, due to notes of fluid accumulation across the body.

Similar to that of *Prox1*^+/−^ mice, surviving *Vegfc*^+/−^ mice have an underdeveloped lymphatic system, presenting lymphatic hypoplasia and lymphedema (7, 16). These findings complement the surrounding discoveries of VEGFR-3 function during early development. Interestingly, VEGF-D was unable to compensate for VEGF-C during embryonic lymphatic development, suggesting the necessity of VEGF-C in promoting Prox1-expressing LEC migration and thereby lymph sac formation (90). Given the importance of VEGFR-3 in the proper organization of blood vessels during early embryogenesis, it suggests that VEGF-D is more integral to cardiovascular development. This elucidation of VEGF-C's role in lymphangiogenesis has implicated its roles in cancer progression (91, 92), serving as an effective predictive marker for lymph node metastasis for some cancers (93, 94).

## RAS, An Essential Mediator in Vascular Development

**Figure 1:**
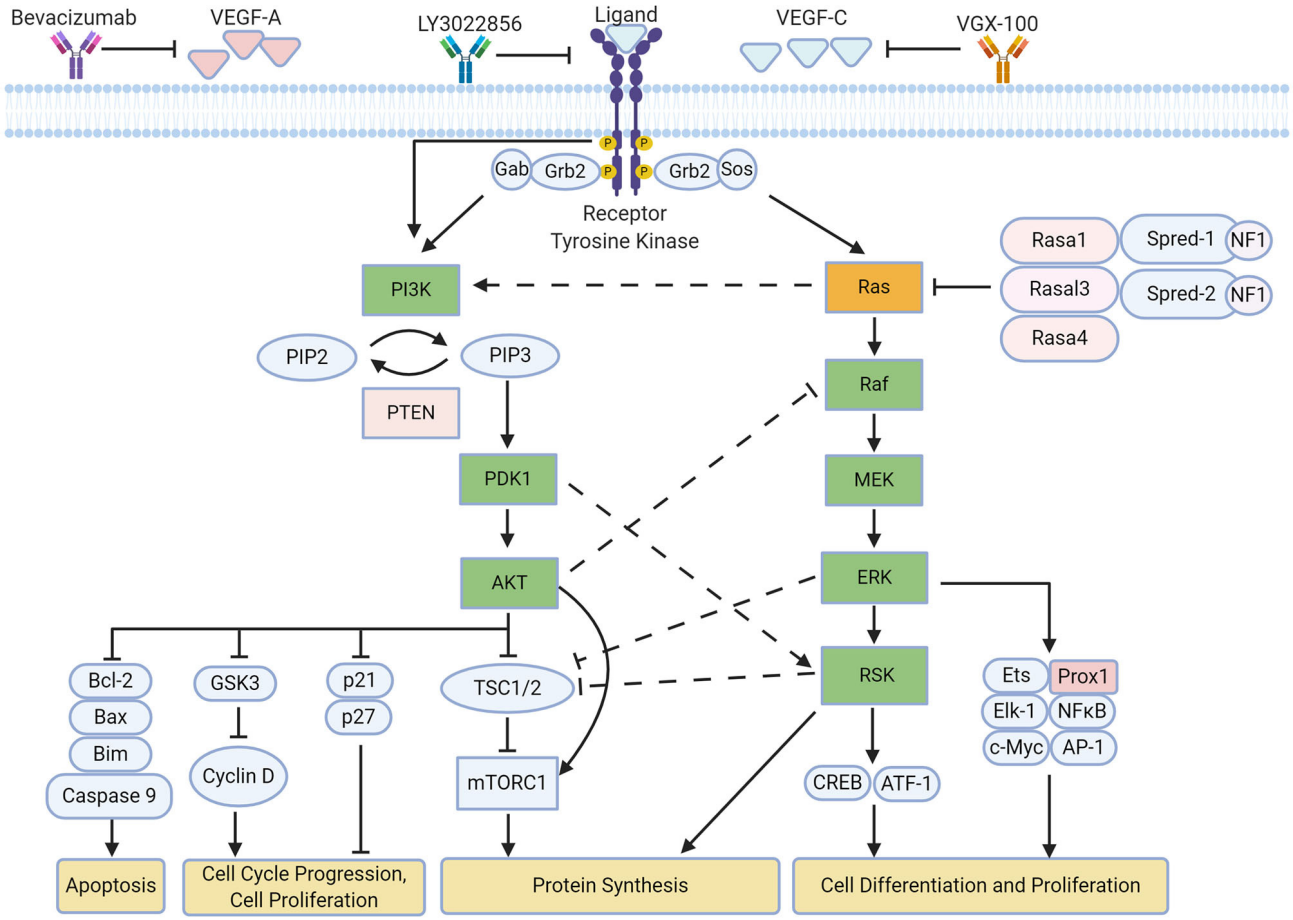
Overview of ERK and AKT pathway on lymphangiogenesis. Both pathways influence different aspects of lymphatic remodeling upon activation of VEGFR-3, leading to downstream phosphorylation of signaling proteins (90). Crosstalk occurs across the pathways primarily as a means of negative regulation (95–97). Current therapies to inhibit these signaling pathways involve the sequestration of VEGFR and its ligands (98–100). Created with BioRender.com.

Lymphangiogenesis is regulated by various signaling cascades mediated by VEGFs/VEGFRs (90) (Figure 1). Further investigation has shown the importance of Ras in modulating multiple signaling pathways following VEGFR activation (101).

In LECs, much of Ras signaling is dependent on VEGFR-3 activation. VEGF-C is primarily received by VEGFR-3, which leads to the receptor dimerization, transphosphorylation and interaction with growth factor receptor-bound protein 2 (GRB2). This leads to the eventual recruitment of guanine nucleotide exchange factors such as the sons of the sevenless (SOS) protein, which activates the small GTPase Ras via GTP binding. This can be inactivated by GTPase-activating proteins (GAPs), which promotes its GTPase activity to hydrolyze GTP to GDP, resulting in an inactive GDP-bound state. Active GTP-bound Ras leads to the activation of multiple pathways including the AKT (Protein Kinase B) and ERK (Extracellular Signal-Regulated Kinase) pathway, which is also referred to as the MAPK (mitogen-activated protein kinase) pathway.

These pathways are heavily involved in promoting survival, proliferation and migration, which are integral to lymphangiogenic sprouting (90, 102). Interestingly, the activation of each pathway is dependent on the dimerization state of VEGFR-3 upon VEGF-C stimulation; the formation of a VEGFR-3/VEGFR-2 complex activates AKT signaling, whereas ERK signaling is activated following VEGFR-3 homodimerization (103). Furthermore, there is crosstalk between the ERK pathway and AKT pathway; phosphorylation of Raf by AKT (also known as Protein Kinase B) leads to the inhibition of the Ras-Raf-MEK-ERK cascade (95), which can be seen in proliferating cells (104). In contrast, phosphoinositide-dependent kinase 1 (PDK1), which is activated by PI3K activity, can phosphorylate a downstream target of the ERK pathway, ribosomal S6 kinase 2 (RSK2), to lead to its full activation (96). This suggests that the activation of multiple pathways may serve to regulate one another.

As the Ras pathways play an essential role in regulating cell cycle, growth, differentiation and survival, their dysregulation leads to severe consequences. This has been well-documented in oncogenesis with their discovery as the first human oncogenes over three decades ago, where it is strongly argued that Ras gain-of-function somatic mutations play a causative role in human tumorigenesis (97). There are three canonical *Ras* genes (*H-Ras, N-Ras, or K-Ras*), which vary in distribution and frequency across different organs/cancers (105); *K-Ras* mutations are present in a majority of pancreatic ductal cancers but uncommon in bladder tumors, where *H-Ras* mutations are likely detected (97).

Further studies revealed the importance of Ras signaling during development, with a collection of unique mutations leading to disorders commonly referred to as “RASopathies.” RASopathies are a class of developmental disorders caused via germline mutations in important regulators of the Ras-ERK pathway (106), though PI3K-AKT signaling participates in this pathophysiology as well (107). This group of disorders include Noonan syndrome, Cranio-facio-cutaneous syndrome (CFCS), and LEOPARD syndrome, which are affiliated with ~20 different disease genes in these pathways but present similar symptoms: congenital heart defects, postnatal proportionate short stature, developmental delay, facial dysmorphism, and so on (107). Additional information regarding these RASopathies such as protein function, chromosomal location and phenotype are excellently summarized in a past review (106). Further investigation has revealed that patients with Noonan or CFCS syndrome had a consistent pattern of bilateral lower limb lymphedema and chylous reflux (108).

Mouse knockout and overexpression studies of Ras have presented lymphatic vascular hypoplasia and hyperplasia, respectively (21). Studies have shown that constitutively active Ras speeds up cell migration and thereby wound healing (109), which would be interesting to investigate in the context of cardiac function following infarction. While Ras has been shown to be invaluable in the regulation of multiple signaling pathways, each Ras isoform plays different roles throughout the body. This is due to Ras isoforms being differentially expressed, with their dysregulation leads to different but related lymphatic vascular disorders (110). For example, Noonan syndrome and CFCS consist of varying K-Ras activating mutations, in which both disorders present scenarios where Ras activity is unable to be negatively regulated to a degree (111, 112). K-Ras mutations are also the most frequent out of the three isoforms in a majority of cancers, such as pancreatic, colon and lung cancer (113). Despite this differential expression, the Ras isoforms share common mutation sites, being at G12, G13, and Q61. The majority of H-Ras missense activating mutations occur at G12, which is integral to Q61 orientation for GAP-promoted GTPase activity and thereby inactivation of Ras (114). The same can be said with K-Ras, where G12 mutations comprise 83% of all K-Ras mutations, whereas N-Ras is predominantly mutated at Q61 (115).

Dysregulation in the negative regulators of Ras has also resulted in developmental defects and abnormal lymphatic vasculature. For example, the knockout of Spred-1 and NF1 result in similar RASopathies, Legius syndrome, and Neurofibromatosis-1, respectively (116). Double knockout studies of Spred-1 and Spred-2 found that these proteins were essential for embryonic lymphangiogenesis, resulting in embryonic death from E12.5–15.5 whilst presenting edema and dilated lymphatic vessels (24). These knockouts resulted in increased ERK phosphorylation and subsequent activation. NF1 knockdown in HUVECs appeared to present the same effects, where cells proliferated at an increased rate and failed to undergo normal branching morphogenesis (117). It was later found that Spred-1 and Spred-2 interact with NF1 to downregulate Ras-GTP levels and subsequent pathway activation; Spred-1 induces plasma membrane localization of NF1, which acts as a GAP on Ras (118). However, Spred-1 has been reported to act in a Ras isoform-specific manner, where Spred-1 prevents K-Ras membrane anchorage but not H-Ras (119). This would suggest the involvement of other regulators in this pathway that have yet to be uncovered. *Rasa1*, which codes for p120-RasGAP, has been shown to be repressed in colorectal cancer, allowing for the increased activation of Ras (120). Loss-of-function *Rasa1* mutations (e.g., A3070T, C2245T) have been found to cause capillary malformation-arteriovenous malformation (CM-AVM) (121, 122), vascular abnormalities that can range from macular staining to abnormal bleeding and life-threatening complications. Systemic loss of *Rasa1* resulted in lymphatic vessel disorders characterized by extensive vessel hyperplasia and leakage, as well as early lethality due to chylothorax (22). Follow-up studies found that *Rasa1* regulates lymphatic vessel valve function, in which LEC apoptosis around these valves explains the leakage defects (123). Further investigation has revealed that *Rasa1* disruption impairs the export of collagen IV from ECs during developmental angiogenesis, which leads to apoptotic death due to endoplasmic reticulum stress (124).

## The AKT Pathway and Lymphangiogenesis

The PI3K-AKT pathway is highly conserved and is controlled through a multistep process. Phosphatidylinositol 3-kinase (PI3K) can be activated by one of three means: the direct binding of PI3K's regulatory subunit p85α by (1) Ras, (2) the scaffolding protein known as growth factor receptor-bound protein 2 (Grb2)-associated binder (GAB), or (3) the receptor tyrosine kinase itself (125). PI3K then phosphorylates phosphatidylinositol (4,5)-bisphosphate (PIP2) to phosphatidylinositol (3-5)-triphosphate (PIP3). This process could be negatively regulated by a PIP3 phosphatase known as Phosphatase and Tensin Homolog (PTEN), whose deletion or mutation leads to can lead tumorigenesis and excessive angiogenesis (126, 127). This PIP3 serves as a docking phospholipid that binds to AKT, allowing PDK1 access and phosphorylate AKT's T308 in the activation loop (128). From there, AKT can phosphorylate a number of targets such as the tuberous sclerosis 1 and 2 (TSC1-TSC2) complex, mammalian target of rapamycin complex 1 (mTORC1) and Caspase 9, thereby promoting protein synthesis and survival (129, 130). In addition to this, PDK1 and mTORC1 can activate ribosomal protein S6 kinase (S6K), which also stimulates protein translation (131, 132).

Aberrations in AKT signaling represent a broad spectrum of human diseases such as cancer, immunological disorders, cardiovascular disease and so on (133). Overactivation of this pathway via AKT hyper-phosphorylation tends to lead to excessive cell growth and division. AKT-mediated signaling was well-known in blood vascular development and was eventually shown to be required for proper lymphatic development; AKT-deficient mice (*Akt1*^−/−^) presented decreases in lymphatic capillary diameter, losses in valves typically present in the collecting lymphatic vessels, and sparse smooth muscle coverage of such vessels (23). Follow-up studies found that the phosphorylation of AKT and ERK1/2 played large roles in VEGF-A/VEGFR-2-mediated lymphangiogenesis, as the inhibition of either protein's upstream kinase led to decreased LEC migration and proliferation (134). These discoveries have opened new avenues for treating lymphatic disease, as AKT's involvement in lymphangiogenesis became clearer. Our lab has gotten involved with these treatments, reporting that 9-cis retinoic acid may be a promising therapeutic agent against secondary lymphedema, as retinoic acid could promote the proliferation, migration and tube formation of LECs via FGFR-signaling and thereby AKT activation (135).

PI3K is composed of four subgroups (class Ia, Ib, II, III), though growth factor receptors primarily activate class Ia, dimeric proteins consisting of a catalytic and regulatory subunit (136). Mutations in both subunits have been shown to impair lymphatic sprouting and maturation. For example, the deletion of *Pik3r1*, which encodes the regulatory isoforms p85α, p55α, and p50α, led to intestinal lymphangiectasia marked with increases in lymphatic endothelial endoglin expression. Interestingly, the effects varied by organ site, as the diaphragm was marked with arrested lymphatics, while the gut showed lymphatics invading the gut (136). Many distinct activating mutations in *PIK3CA*, one of four catalytic isoforms in class I PI3Ks, present mutation hotspots in human tumors (133). In addition to this, constitutively active PIK3CA mutations were found to be expressed in LECs and vascular endothelial cells (VECs) in capillary lymphatic venous malformations, leading to continuously phosphorylated AKT and hyperproliferation of these cells (137). These gain-of-function mutations typically either bypassed PI3K's requirement to interact with Ras (H1047R) or disrupted regulatory subunit interface (E542K/E545K), leading to the pathway's hyperactivation (138, 139). Given this pathway's involvement in both lymphangiogenesis and angiogenesis, many vascular overgrowth disorders are associated with these mutations (140).

Recently, the combined treatment of VEGF-C trap and rapamycin, but neither treatment alone, were found to promote lesion regression in PIK3CA^H1047R^-driven lymphatic malformations, through lymphatic vasculature regression and blockage of LEC proliferation (141). This highlights the importance of combination therapy on both upstream and downstream elements of a mutant effector molecule, suggesting the combined impact of other signaling proteins in the generation of these physiological changes. Other studies found that PTEN knockouts in endothelial cells result in increased cancer susceptibility and embryonic lethality, due to aberrant differentiation, hyperproliferation and disorganized vasculature (142, 143).

Interestingly, the regulation of lymphangiogenesis through PI3K is not entirely restricted to Ras-mediated activation. VEGF-C can induce PI3K-dependent AKT activation through VEGFR-3, where VEGFR-3 forms a complex with the PI3K regulatory subunit p85 (144). As seen with other receptor tyrosine kinases, insulin receptor substrates (IRS) such as GAB are typically recruited as adaptor proteins for PI3K regulation and activation (145), suggesting that they may be recruited by VEGFR-3. In alignment with these reports, IRS blockade was found to inhibit lymphangiogenesis by reducing proliferation and VEGF-A expression in LECs (146).

AKT modulates multiple cellular functions through inhibitory and activating phosphorylation events. AKT is well-known in promoting cell growth through inhibiting TSC2 and thereby activating mTORC1, which initiates translation and ribosome biogenesis (145, 147). In addition to this, AKT can inactivate cyclin-dependent kinase inhibitors such as p21 and p27, allowing for cell cycle progression (148). AKT can also inhibit apoptosis by blocking proapoptotic protein function of Bcl-2, Bax, and Bim proteins (145).

## The Extracellular Signal-Regulated Kinase (ERK) Pathway and Lymphangiogenesis

The ERK/MAPK pathway relies on the binding of growth factors to induce a series of phosphorylation cascades, which begins with the activation of Raf by GTP-bound Ras. From there, Raf phosphorylates MEK, which phosphorylates ERK, allowing it to phosphorylate many downstream targets in both the cytoplasm and nucleus. This system provides opportunities for feedback regulation as well as signal amplification with each subsequent phosphorylation event.

Gain-of-function mutations in the Ras/Raf signaling cascade present lymphatic defects such as lymphangiectasia, which is prominent in patients with Noonan and LEOPARD syndrome (149, 150). RAF1 in particular has been recognized as a major effector whose gain-of-function mutations cause Noonan and LEOPARD syndromes, emphasizing the importance of the ERK pathway activation in developmental disorders (151, 152).

Following these discoveries, many studies investigated the dysregulation of ERK pathway through loss- and gain-of-function mutants in upstream and downstream elements of Ras. VEGFR-3 bearing deletions in the cytoplasmic domain at tyrosines Y1226/7 prevented ERK phosphorylation and lymphatic sprouting, which were rescued with autonomous ERK activation (153). Indeed, it was found that hyperactivation of the ERK pathway resulted in increased LEC proliferation or fate specification, where gain-of-function mutant RAF1^S259A^ embryos led to lymphangiectasia (154). Loss of negative regulation has presented similar results; the loss of Foxc1 and Foxc2 presented decreases in GAPs encoded by *Rasa4* and *Rasal3*, resulting in increased ERK activation and thereby LEC proliferation and enlarged lymphatic vessels (20). Recently, ERK pathway inhibition has shown some promise as treatment for lymphatic anomaly, where an advanced anomalous lymphatic disease patient possessing a gain-of-function ARAF recurrent mutation was unresponsive to mTOR inhibition but not to MEK inhibition, marked by decreased lymphedema and improvement in pulmonary function (155). Together, these studies make it evident that the ERK pathway is vital to lymphatic remodeling.

ERK possesses numerous downstream targets, including other kinases, transcription factors and so on. Many of these transcription factors that are directly activated by ERK, such as activator protein 1 (AP-1), c-Myc, and erythroblastosis virus oncogene homolog 1 (Ets-1), were discovered as proto-oncogenes (156). S6K has also been recognized as a target of ERK in cardiomyocytes (157). Kinases downstream of ERK, such as RSKs, have been found to activate CREB and cyclic AMP-dependent transcription factor 1 (ATF-1), transcription factors that are also implicated in cell transformation (156, 158). Furthermore, ERK and RSK can inhibit TSC1-TSC2 complex activity via phosphorylation (159).

Given the range of targets, it is difficult to ascertain which of ERK's downstream targets are integral to lymphatic sprouting and differentiation. Increases in ERK signaling through ectopic expression and mutant Raf presented increases in Prox1 and other LEC-specific genes, highlighting the broad induction of lymphatic fate determination (154). This finding is consistent with the literature, as Ras-ERK signaling leads to the activation of many transcription factors through direct phosphorylation or the subsequent activity of other downstream effectors.

## VEGFR-3, Signaling Pathways and PROX1 Interaction: Feedback Loops

LECs require the stable expression of Prox1 to maintain their identity; Prox1 siRNA-treated LECs revert to a BEC phenotype (160). Due to this constant expression, Prox1 is heavily used as a lymphatic marker. Its transcriptional activity is frequently observed to determine the effects of signaling pathways, as increased Prox1 leads to the upregulation of other lymphatic markers and ultimately lymphangiogenesis. While VEGFR-3 signal transduction has been recognized as one of the major pathways involved in lymphangiogenesis, the mechanisms by which ERK and PI3K may be coordinating Prox1 activity remains unclear.

Several studies have suggested that Prox1 participates in a number of positive feedback loops by promoting the transcription of key signaling proteins and other transcription factors that target the Prox1 promoter and enhancer regions. For example, HoxD8 levels are significantly higher in LECs than BECs, as its transcription is upregulated by Prox1 and positively regulates Prox1 transcription in return (54). Chicken ovalbumin upstream promoter-transcription factor 2 (Coup-TFII), which was shown to act as a coactivator for Prox1, is also required for the initiation and maintenance of Prox1 expression in LECs, as Coup-TFII can directly bind to a conserved binding site in a regulatory region ~9.5 kb upstream of the Prox1 ORF (161). However, it has been suggested that ERK signaling is capable of inducing Prox1 expression in the absence of Coup-TFII (154).

In LECs, Prox1 has also been shown to interact with multiple transcription factors for its self-regulation as well as expression of VEGFR-3. Nuclear hormone receptor Coup-TFII is required for the initiation and maintenance of Prox1 expression of LECs (161) and acts as a coactivator of Prox1 to promote FGFR3 and VEGFR-3 transcription (162). The transcription of potent regulator APN in angiogenesis is induced by Ras-mediated phosphorylation of Ets-2 (163), which also interacts with Prox1 to bind to the VEGFR-3 promoter (164).

Given the role of VEGFR-3 in lymphangiogenesis, the sustained expression of this receptor in LECs is integral to its maintenance. Interestingly, Prox1 has been shown to maintain LEC identity through a Prox1-Vegfr3 feedback loop, where the downregulation of either one results in the downregulation of the other (165). The activation of this signaling pathway ultimately induces Prox1 transcription. Ras/ERK signaling was found to mediate p300 recruitment through Ets activation, leading to histone acetylation on the *Vegfr3* gene in LECs (166). Given Prox1's interaction with Ets-2 (165), Prox1 may be involved with this p300 recruitment to enable VEGFR-3 transcription.

Research on cancer metastasis have implicated the potential roles of Prox1 in the regulation VEGF-C autocrine signaling. It was discovered that CCAAT-enhancer binding protein-delta (C/EBP-δ) upregulates VEGF-C and VEGFR-3 expression in LECs in lung cancer, where hypoxia could induce this transcription factor's expression (167). Following this discovery, cultured oral squamous cell carcinoma cell lines reported increased levels of Prox1 in the “highly-metastatic” lines, which were found to activate VEGF-C expression (34). Together, these findings suggest the cooperation of Prox1 and C/EBP-δ to modulate VEGFR-3 signaling in LECs.

## Problems with Current Anti-Angiogenesis Therapies

Inhibition of angiogenesis has been shown as a viable anti-cancer therapy, with VEGFs and VEGFRs as a major target for these treatments. These include the anti-VEGF-A monoclonal antibody bevacizumab and broad-range receptor tyrosine kinase inhibitor sunitinib, the former of which being approved for combination use with chemotherapy for cancer (168, 169). Small molecule inhibitors such as Sorafenib and Sunitinib have also been used to target a broad range of receptor tyrosine kinases in hopes of overcoming multiple pathways. Many of the existing clinical trials are currently investigating the effects of these drugs in combination with one another or other current therapies, in hopes of increasing patient outcomes.

Despite these advances, many cancers have achieved resistance to anti-angiogenesis therapies through adaptive mechanisms, such as an upregulation of alternative angiogenic factors, recruitment of vascular progenitor cells, and increase in pericyte coverage, to name a few (170). Even bevacizumab, which is considered as one of the most effective anti-angiogenic drugs to date, yields only modest improvements, adding only around 5 months of progression-free survival (PFS) in patients with metastatic colorectal cancer (98).

With these limited results, researchers have also looked targets further downstream of these pathways. There are MEK and mTOR inhibitors that have been approved or are undergoing trials for clinical use by the US Food and Drug Administration (FDA), though it appears that their impact only adds a few months to PFS. Trametinib and Dabrafenib are MEK inhibitors used in combination for metastatic melanoma that presented a BRAF V600 activating-mutation, adding around 2.2 months of PFS when compared to dabrafenib alone (Clinical Trial NCT01584648) (171). mTOR inhibitors such as Everolimus were found to increase PFS by around 2.1 months for patients with metastatic renal cell carcinoma that has progressed from receptor-targeting drugs such as sunitinib (Clinical Trial NCT00510068) (172).

As characterized in previous reviews, there are no FDA-approved selective agents that specifically suppress lymphangiogenesis (173), despite the growing evidence on their contributions to cancer and other diseases. Anti-lymphangiogenesis therapies are following the same approach as anti-angiogenesis therapies, targeting the key receptors and growth factors of the lymphatic system. A phase I study was completed for anti-VEGFR-3 monoclonal antibody, IMC-3C5, against solid tumors and colorectal cancer. They were able to set a tolerable dose, but only saw minimal anti-tumor activity so far (Clinical Trial NCT01288989) (99). There was also a phase I study for anti-VEGF-C monoclonal antibody, VGX-100, for combination usage with bevacizumab against advanced solid tumors (Clinical Trial NCT01514123) (100). While these therapies may be promising, they may encounter the same levels of resistance that were seen with current anti-angiogenesis therapies, highlighting the need for a different group of targets.

## Post-Translational Modifications and Prox1: A Promising Frontier on Lymphangiogenesis

As transcription factors have the capability of reconfiguring cellular physiology and function, their regulation through molecular modification is essential to proper function. Post-translational modifications (PTMs) play a large role in modulating protein stability, protein-protein interaction, DNA-binding, subcellular localization and so on (174). Many of these PTMs occur as individual, isolated events to dictate some aspect of transcription factor function, though some PTMs are sequentially linked, enabling (or inhibiting) one another (175).

Despite this, modifications on Prox1 remain poorly understood. To date, only the effects of sumoylation on Prox1 at two separate lysine sites have been investigated, though other putative sumoylation sites have been recognized (176). These two sites, Lys353 (K353) and Lys556 (K556), were revealed using SUMOplot analysis. In addition to this, both sites are highly conserved in different species, including humans, mice, chicken, and zebrafish (177). Sumoylation of Prox1 K556 by small ubiquitin-related modifier 1 (SUMO-1) enables Prox1 binding to the VEGFR-3 promoter to upregulate transcription (177). Without this sumoylation, VEGFR-3 signaling was significantly diminished and led to impaired sprouting. This may in part explain the feedback loop between Prox1 and VEGFR3, though it remains unknown how VEGFR3 signaling impacts Prox1. Further investigation found that the ectopic expression of K556R mutant Prox1 could not induce a lymphatic phenotype in HUVECs (177), as compared to the previous studies with wildtype Prox1 (54). These findings highlight the importance of sumoylation on Prox1 functionality.

PTMs on Prox1 also impact its corepressor activity; sumoylation at K353 inhibits Prox1 interaction with HDAC3 in LECs, thereby downregulating Prox1's corepressor activity (176). Given this finding, sumoylation may be vital to the previously mentioned roles of Prox1 in the liver, given its interaction with HDAC3, HDAC2, and LSD1.

**Figure 2:**
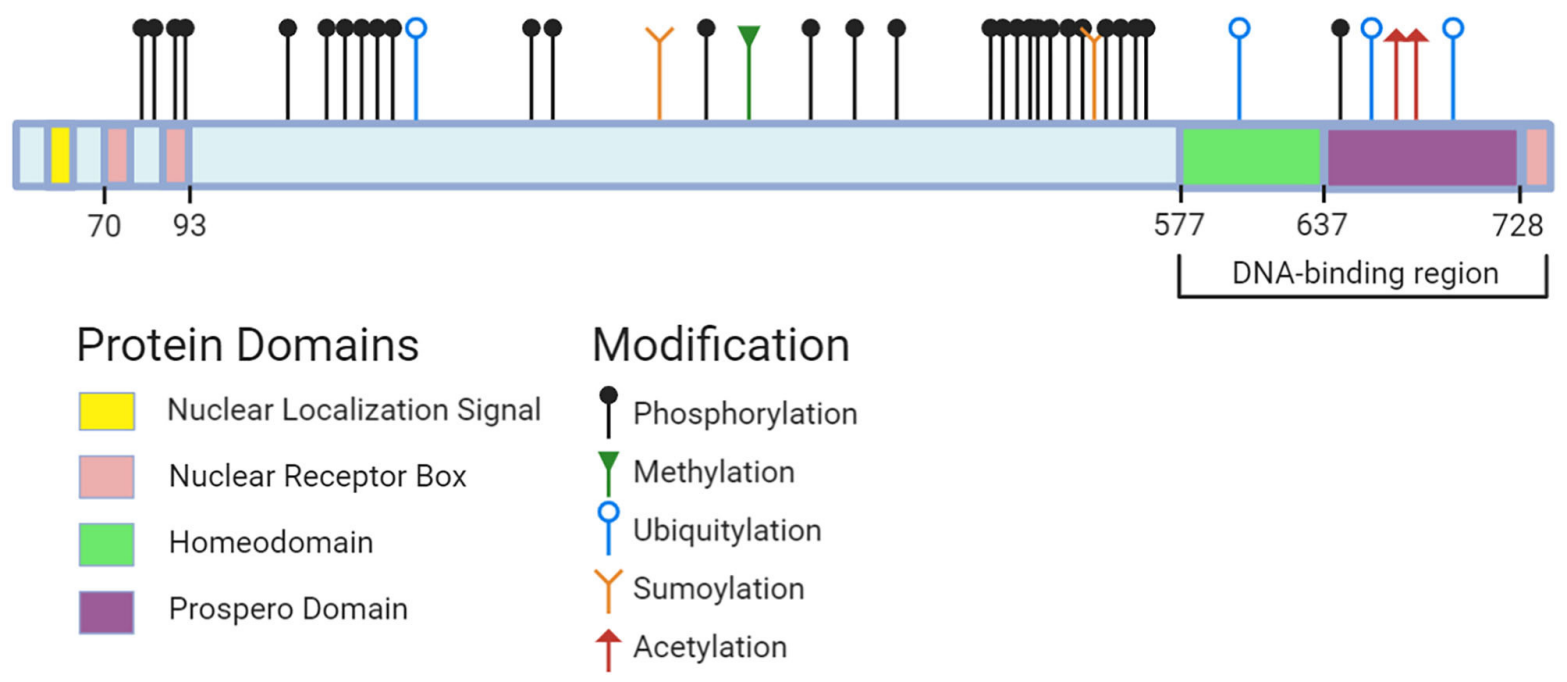
Prox1 protein domains and potential sites for modification. Data on sites across human, mouse, and rat Prox1 were pulled from PhosphoSitePlus (accessed October 1st, 2020). The following modification types and associated residues are: (1) Methylation (R392); (2) Sumoylation (K353, K556); (3) Acetylation (K707, K708); (4) Ubiquitylation (K212, K611, K700, K716); (5) Phosphorylation (S79, Y80, T91, T94, S142, S177, S179, S181, S197, S199, S291, S295, S372, S432, S472, S495, S511, S514, T518, S529, S530, S539, S545, S553, K557, K569, Y571, S574, T650). Created with BioRender.com.

Not much is known about the roles of other Prox1 PTMs, which include acetylation, methylation, ubiquitylation and phosphorylation. Comprehensive online resources such as PhosphoSitePlus have compiled data sets from journal articles to identify modifiable sites on Prox1 (Figure 2). Acetylation sites have been detected in the homeo-prospero domain using liquid chromatography mass spectrometry, suggesting this modification's potential involvement in DNA-binding activity. Several phospho-sites spanning Prox1 have also been identified using mass spectrometry-based approaches (178, 179). Prox1 methylation has not been documented through these approaches. In short, the roles of these modifications and the enzymes responsible remain unknown.

## Concluding Remarks

Lymphangiogenesis is a complex process that is heavily tied with VEGFR-3 signaling and Prox1 activity. The feedback loops that these proteins manage are essential to not only this sprouting capability but sustaining of LEC fate. RASopathies have highlighted the importance of the ERK and PI3K pathways in lymphatic development, where many of these genetic syndromes are attributed to gain-of-function mutations in their upstream elements. Consequences of hyperactivation with these pathways include proliferation as well as cell death, in which these seemingly paradoxical effects result in leaky vasculature.

The roles of these signaling pathways have been well-characterized in lymphatic disease and cancer, suggesting the benefits of dual pathway inhibition. The same could be said about stimulation, given the lymphatic system's roles in wound healing and resolution of inflammation. It would be crucial to target both the activity of negative and positive regulators throughout these pathways, as cancer may overcome the targeting of either group through the overexpression of repression of the other. It may be worthwhile to investigate whether this combinatorial approach leads to synergistic or additive effects on lymphangiogenesis.

These signal transduction events modulate transcription factor activity with Prox1 or possibly Prox1 itself. However, there remains some disconnect between these signaling pathways and Prox1; we lack information on the impact and causes of post-translational modifications of Prox1. As seen with sumoylation, these modifications could significantly impact Prox1 DNA-binding and coactivator/corepressor activity, ultimately influencing lymphangiogenesis. Given Prox1's vital role in a number of organ systems, it would be imperative to see how these signaling pathways could lead to these modifications and impact its lymphangiogenic ability. This knowledge will inform us of potential therapeutic targets that may overcome the resistances seen with VEGF and VEGFR targeting.

## Author Contributions

KB drafted and wrote the literature review. KB and Y-KH contributed to the review and final approval for the literature review. All authors contributed to the article and approved the submitted version.

## Conflict of Interest

The authors declare that the research was conducted in the absence of any commercial or financial relationships that could be construed as a potential conflict of interest.
